# Author Correction: P53 and mTOR signalling determine fitness selection through cell competition during early mouse embryonic development

**DOI:** 10.1038/s41467-018-05718-z

**Published:** 2018-08-02

**Authors:** Sarah Bowling, Aida Di Gregorio, Margarida Sancho, Sara Pozzi, Marieke Aarts, Massimo Signore, Michael D. Schneider, Juan Pedro Martinez-Barbera, Jesús Gil, Tristan A. Rodríguez

**Affiliations:** 1 British Heart Foundation Centre for Research Excellence, National Heart and Lung Institute, Imperial Centre for Translational and Experimental Medicine, Imperial College London, Hammersmith Hospital Campus, Du Cane Road, London, W12 0NN UK; 2 Cell Proliferation Group, MRC London Institute of Medical Sciences (LMS), Du Cane Road, London, W12 0NN UK; 3 Cell Proliferation Group, Institute of Clinical Sciences (ICS), Faculty of Medicine, Imperial College London, Du Cane Road, London, W12 0NN UK; 4 Developmental Biology and Cancer Programme, Newlife Birth Defects Research Centre, UCL Great Ormond Street Institute of Child Health, 30 Guilford Street, London, WC1N UK

Correction to: *Nature Communications*; 10.1038/s41467-018-04167-y; published online 02 May 2018.

The original version of this Article contained an error in the spelling of Juan Pedro Martinez-Barbera, which was incorrectly given as Juan Pedro Martinez Barbera. This error has now been corrected in both the PDF and HTML versions of the Article.

