# Supplementary material for: P53 and mTOR signalling determine fitness selection through cell competition during early mouse embryonic development
Source: Nat Commun. 2018 May 2;9:1763. doi: 10.1038/s41467-018-04167-y (PMC5932021; doi:10.1038/s41467-018-04167-y)
Supplement: Supplementary file 1 — Supplementary Information [file 41467_2018_4167_MOESM1_ESM.pdf]

Supplementary information

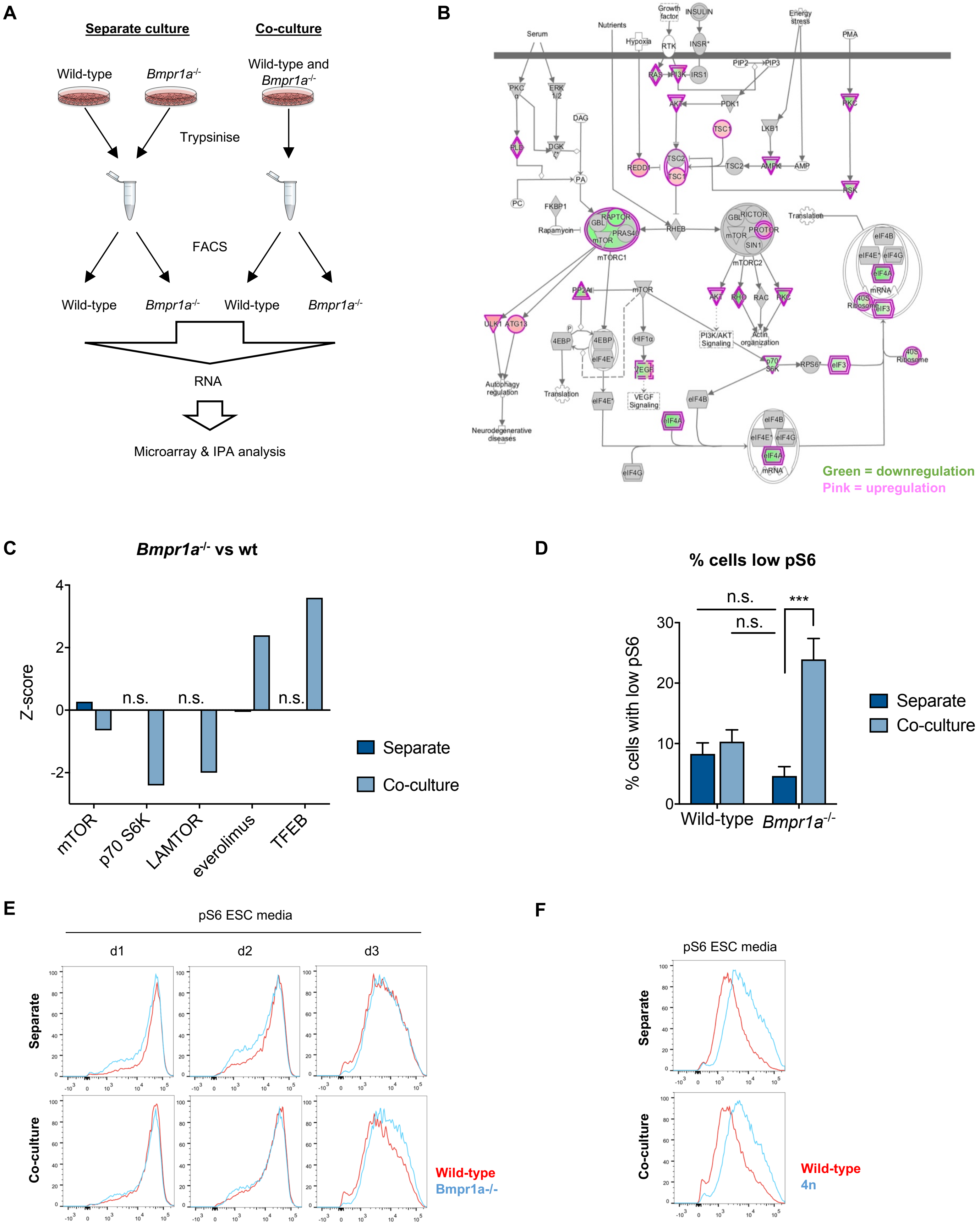

Supplementary Figure 1. Further analysis of mTOR activation during competition.

A. Schematic of the experimental setup, whereby cells were cultured for three days in N2B27 media before FACS, RNA collection, and gene expression analysis by microarray. B. Diagrammatic heat-map revealing mTOR pathway components differentially regulated in *Bmpr1a*$^{-/-}$ compared to wild-type cells in competitive co-cultures. Green denotes pathway components which are downregulated; pink denotes pathway components which are upregulated. The schematic canonical pathway diagrams were generated through the use of IPA (QIAGEN Inc., https://www.qiagenbioinformatics.com/products/ingenuity-pathway-analysis). C. IPA-derived Z-score of mTOR and related pathways in *Bmpr1a*$^{-/-}$ compared with wild-type cells in separate and co-culture. D. Percentage of cells with low pS6 staining in wild-type and *Bmpr1a*$^{-/-}$ cells cultured separately and together for 3 days in N2B27. ***, $p<0.005$; ANOVA and Tukey's post-hoc test. E. Flow cytometry analysis of pS6$^{S240/244}$ in wild-type and *Bmpr1a*$^{-/-}$ cells over three days in ESC media, where pluripotency in maintained and competition is blocked. F. Flow cytometry analysis of pS6$^{S240/244}$ in wild-type and 4n cells at day 3 in ESC media. n=3 for all studies.

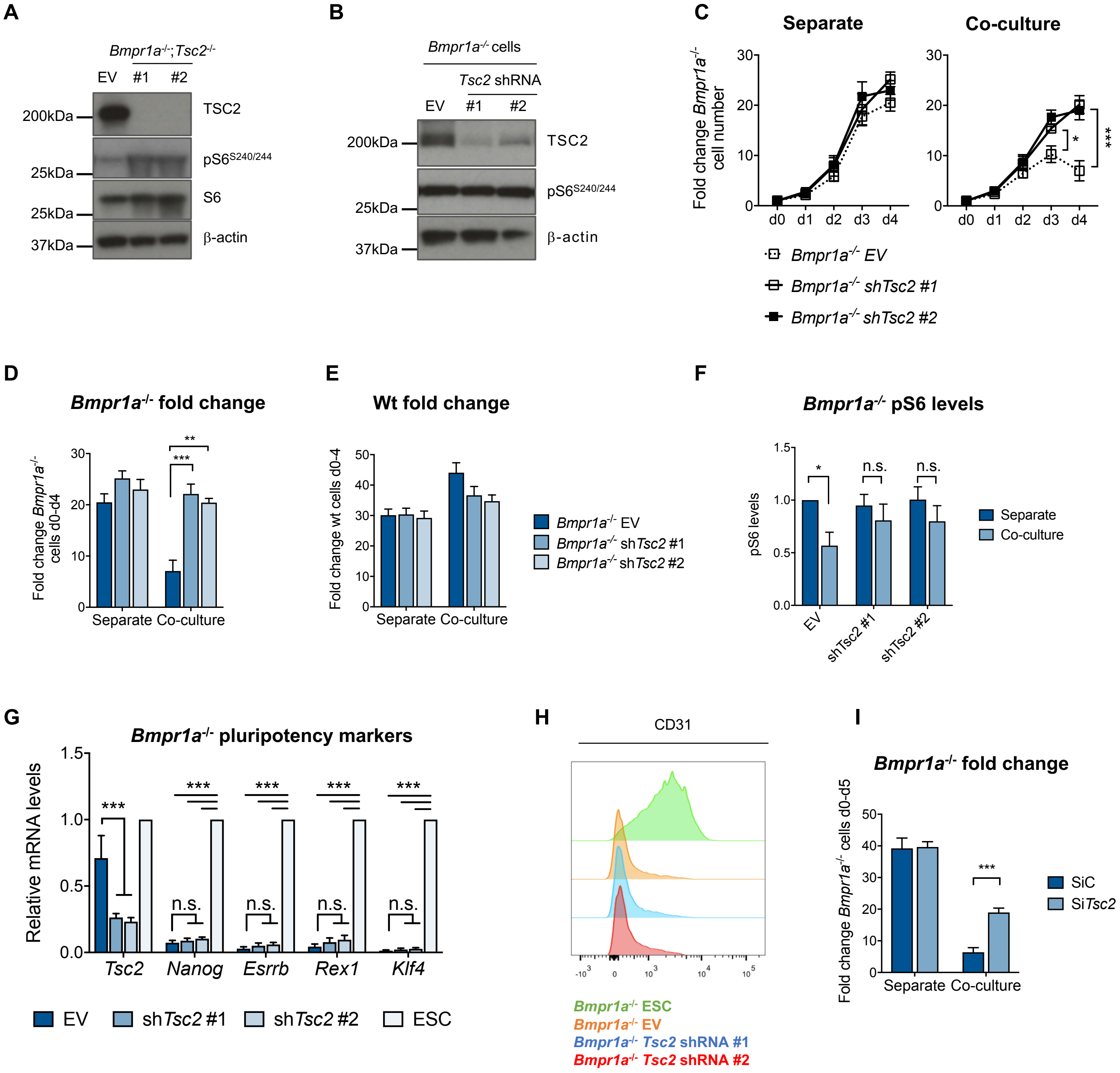
A
Bmpr1a-/-;Tsc2-/-
EV #1 #2
200kDa
25kDa
25kDa
37kDa
TSC2
pS6S240/244
S6
β-actin
B
Bmpr1a-/- cells
Tsc2 shRNA
EV #1 #2
200kDa
25kDa
37kDa
TSC2
pS6S240/244
β-actin
C
Separate
Co-culture
Fold change Bmpr1a-/- cell number
d0 d1 d2 d3 d4
Bmpr1a-/- EV
Bmpr1a-/- shTsc2 #1
Bmpr1a-/- shTsc2 #2
D
Bmpr1a-/- fold change
Fold change Bmpr1a-/- cells d0-d4
Separate Co-culture
E
Wt fold change
Fold change wt cells d0-4
Separate Co-culture
Bmpr1a-/- EV
Bmpr1a-/- shTsc2 #1
Bmpr1a-/- shTsc2 #2
F
Bmpr1a-/- pS6 levels
pS6 levels
n.s.
n.s.
EV
shTsc2 #1
shTsc2 #2
Separate
Co-culture
G
Bmpr1a-/- pluripotency markers
Relative mRNA levels
n.s.
Tsc2 Nanog Esrrb Rex1 Klf4
EV shTsc2 #1 shTsc2 #2 ESC
H
CD31
Bmpr1a-/- ESC
Bmpr1a-/- EV
Bmpr1a-/- Tsc2 shRNA #1
Bmpr1a-/- Tsc2 shRNA #2
I
Bmpr1a-/- fold change
Fold change Bmpr1a-/- cells d0-d5
Separate Co-culture
SiC
SiTsc2

Supplementary Figure 2. mTOR pathway hyper-activation rescues elimination of BMP defective cells independently of differentiation.

A. TSC2 and pS6 levels in *Bmpr1a*$^{-/-}$ cells and *Bmpr1a*$^{-/-}$; *Tsc2*$^{-/-}$ cells where *Tsc2* has been mutated by CRISPR or (B) inhibited using two independent shRNAs. C. Growth curves of *Bmpr1a*$^{-/-}$ cells with or without *Tsc2* shRNA inhibition in separate and co-culture with wild-type cells. D. Fold change in *Bmpr1a*$^{-/-}$ and *Bmpr1a*$^{-/-}$; *Tsc2*^{shRNA} (two independent clones) cell numbers between d0-d4 when cultured alone (separate) or with wild-type cells in N2B27. E. Fold change in wild-type cell numbers between d0-d4 when cultured separately or co-cultured with *Bmpr1a*$^{-/-}$ or *Bmpr1a*$^{-/-}$; *Tsc2*^{shRNA} cells. F. Levels (median fluorescence) of pS6$^{S240/244}$ analysed by flow cytometry in wild-type and *Bmpr1a*$^{-/-}$ cells infected with viruses expressing control or *Tsc2*-targetting shRNA. G. *Bmrp1a*$^{-/-}$ cells were infected with control and *Tsc2*-targetting shRNAs, cultured for three days in N2B27, and expression levels of pluripotency genes *Nanog*, *Esrrb*, *Rex1* and *Klf4* were assessed by qPCR and compared *Bmpr1a*$^{-/-}$ cultured in pluripotent conditions. H. Levels of cell-surface pluripotency marker CD31 were assessed by flow cytometry in pluripotent and differentiating *Bmpr1a*$^{-/-}$ cells infected with control or *Tsc2*-targetting shRNAs. I. Fold change over d0-d5 in *Bmpr1a*$^{-/-}$ cells in the presence of control (SiC) or *Tsc2*-targetting siRNA (Si*Tsc2*). n=3 for all studies. Error bars denote SEM. **p<0.01 and *** p<0.005; ANOVA and Tukeys post-hoc test (C-G and I).

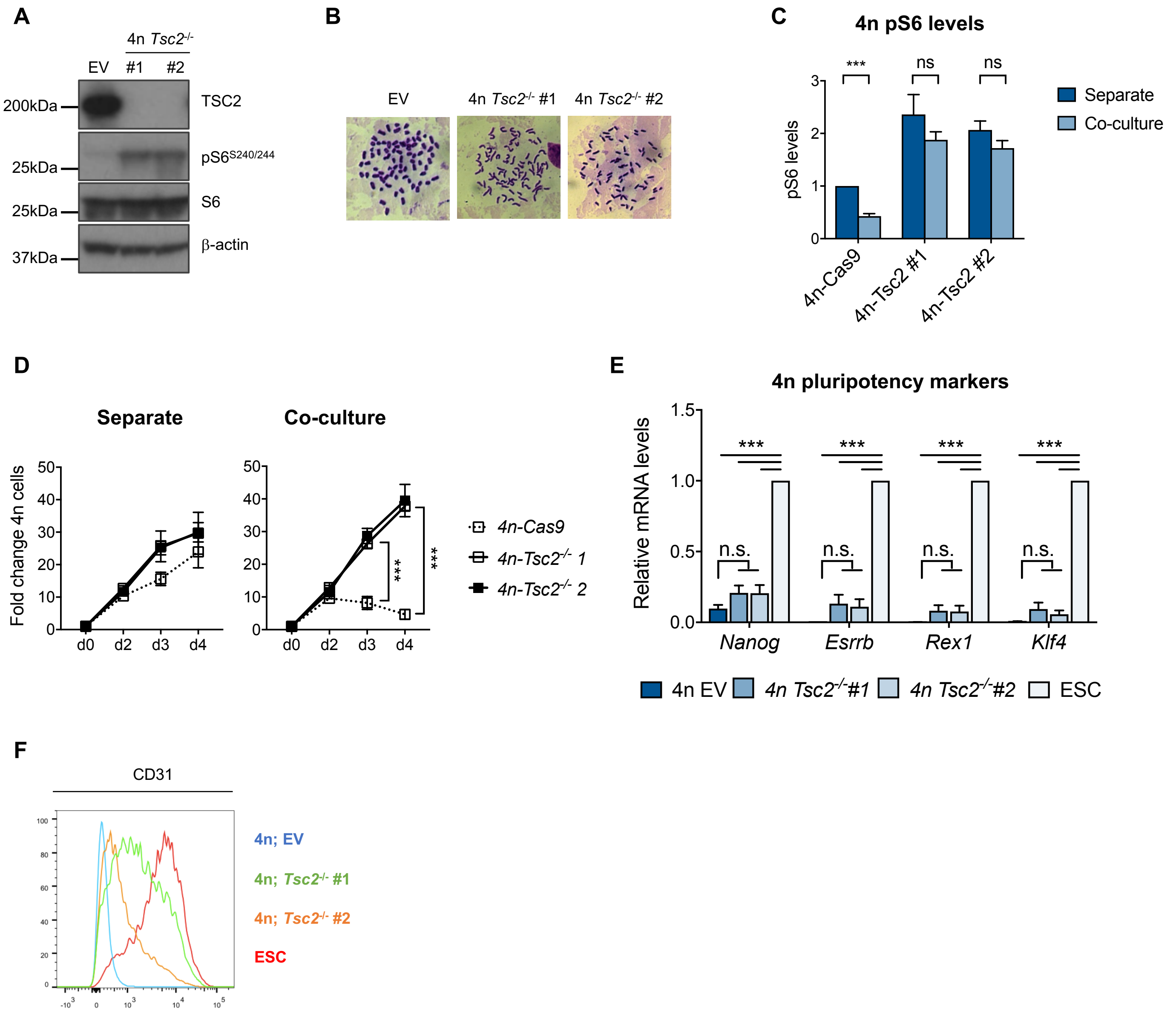

Supplementary Figure 3. Loss of *Tsc2* rescues 4n cell elimination during competition independently of differentiation.

A. Two 4n clones targeted by CRISPR and lacking TSC2 were picked for competition analysis. B. Karyotypic analysis of 4n-*Tsc2*$^{-/-}$ cells. C. Levels (median fluorescence) of pS6$^{S240/244}$ analysed by flow cytometry in wild-type and control/ *Tsc2*$^{-/-}$ 4n cells. D. Fold change in wild-type cells from d0-d4 when cultured alone and in the presence of 4n-Cas9 and the two 4n-*Tsc2*$^{-/-}$ clones. E. Expression levels of pluripotency genes *Nanog*, *Esrrb*, *Rex1* and *Klf4* in control and *Tsc2*$^{-/-}$ 4n cells cultured in N2B27 for 3 days were compared to pluripotent 4n cells by qPCR. Error bars denote SEM. F. Levels of cell-surface pluripotency marker CD31 were assessed by flow cytometry in pluripotent and differentiating control and Tsc2-knockout 4n cells. *** $p<0.005$; ANOVA and Tukeys post-hoc test (C,D,E).

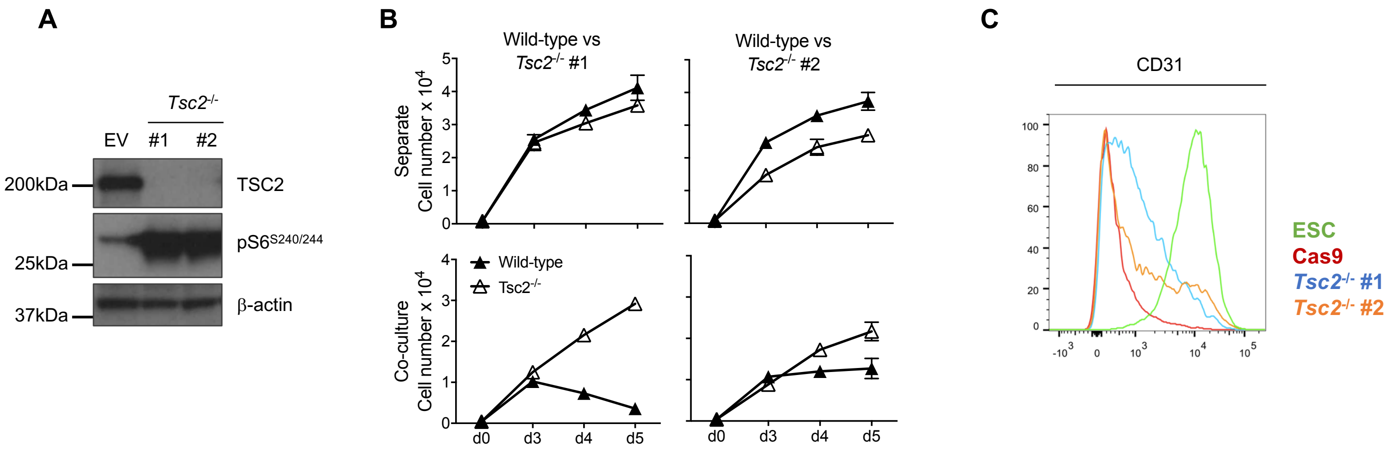

Supplementary Figure 4. Analysis of super-competition mediated by *Tsc2* deletion.

A. Two wild-type clones targeted by CRISPR and lacking TSC2 were picked for competition analysis. B. Growth curves of wild-type and two *Tsc2*$^{-/-}$ cell clones cultured together and separately for 5 days. C. Levels of cell-surface pluripotency marker CD31 were assessed by FACS in pluripotent and differentiating control and *Tsc2*$^{-/-}$ cells. n=3 for all studies.

A

Upstream Regulator	Molecule Type	Predicted Activation State	Activation z-score	p-value of overlap
**TP53**	transcription regulator		0.849	6.73E-33
**MYC**	transcription regulator	Inhibited	-2.846	2.59E-18
**L-dopa**	chemical - endogenous mammalian		-1.895	3.87E-18
**beta-estradiol**	chemical - endogenous mammalian	Inhibited	-3.534	1.93E-17
**ERBB2**	kinase		0.097	2.55E-17
**ESR1**	ligand-dependent nuclear receptor		-1.12	2.90E-17
**TGFB1**	growth factor		-1.259	6.87E-15
**HNF4A**	transcription regulator	Activated	2.042	8.99E-15
**NUPR1**	transcription regulator	Activated	2.397	2.74E-13
**PTEN**	phosphatase		1.194	6.53E-13

B

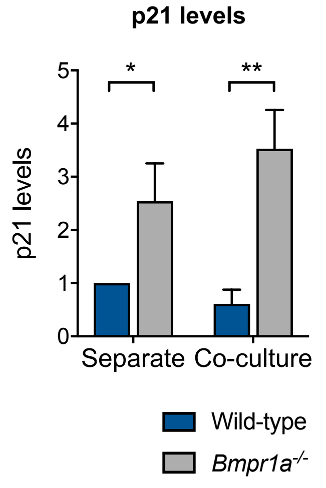

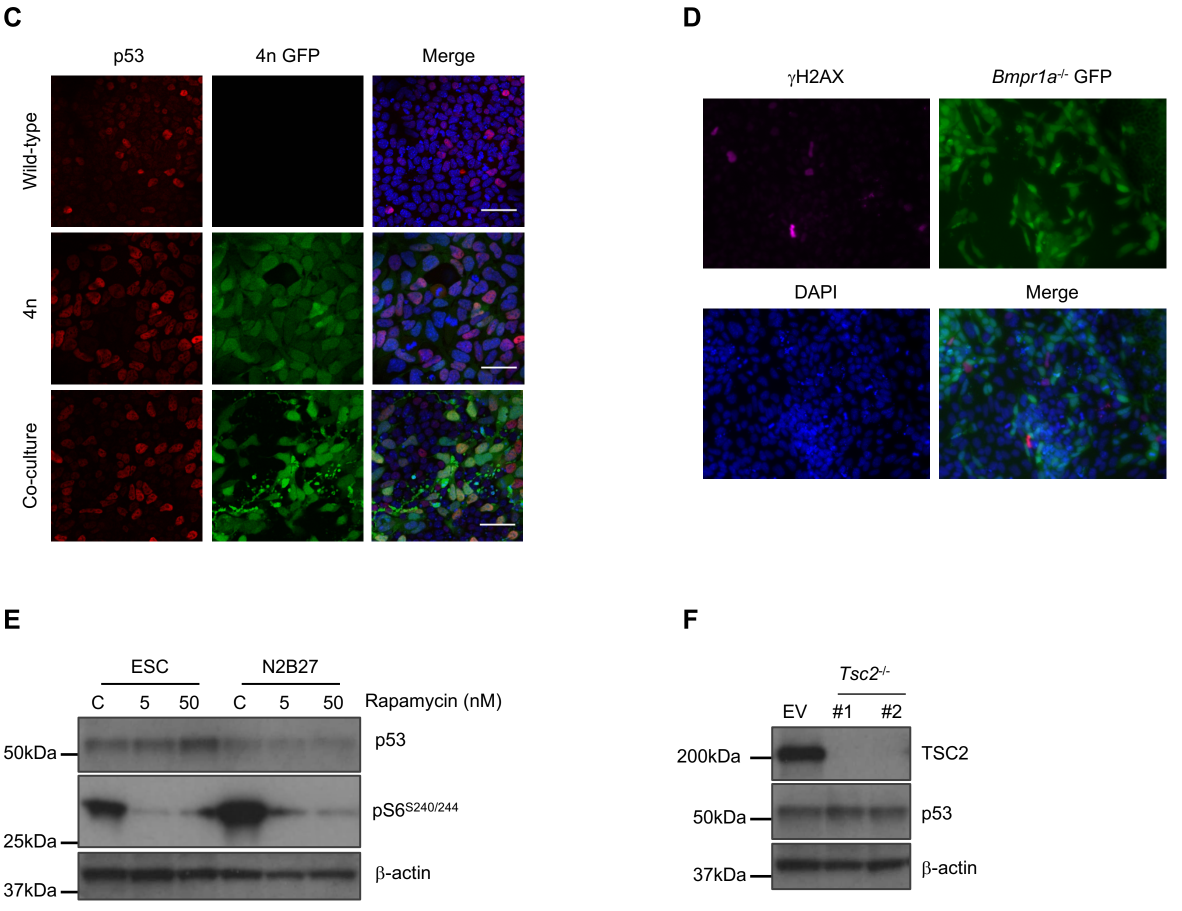

Supplementary Figure 5. Further analysis of p53 levels and possible activators in competition.

A. List of top 10 upstream regulators ranked by *p*-value with differential expression in *Bmpr1a*$^{-/-}$ cells compared with wild-type cells during competition. B. Levels of p21 expression in wild-type and *Bmpr1a*$^{-/-}$ cells in separate and co-culture assessed by qPCR. C. Levels of p53 in wild-type and 4n cells cultured separately and together were assessed by immunofluorescence. Scale bar=50μm. D. Protein levels of DNA double strand break marker gamma-H2AX in co-cultured wild-type and *Bmrp1a*$^{-/-}$ cells were assessed by immunofluorescence. E. Cells were treated with rapamycin for 6 hours and levels of p53 were assessed by Western blot. F. Levels of p53 in *Tsc2*$^{-/-}$ cells were assessed by Western blot. n=3 for all studies.

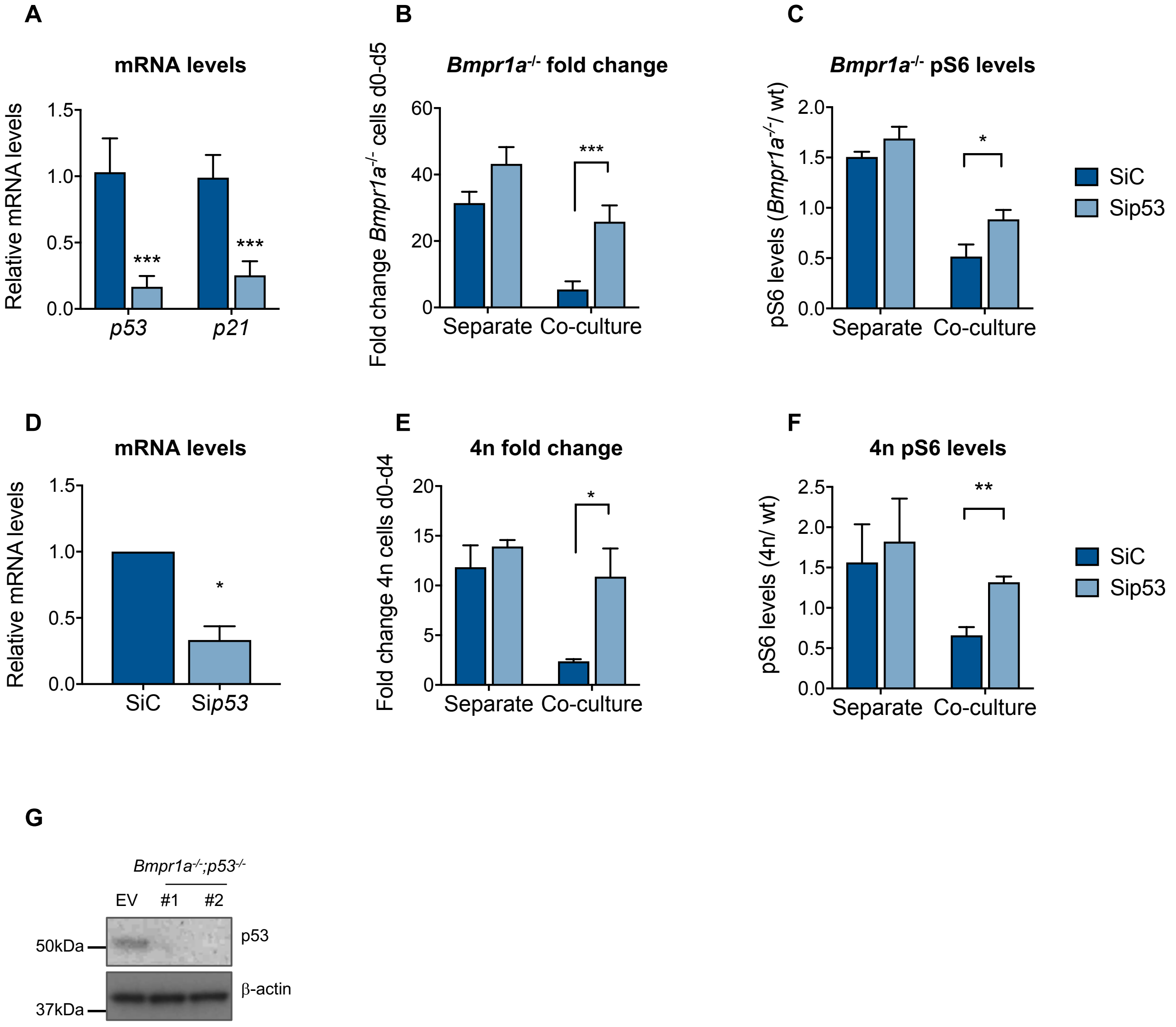

Supplementary Figure 6. *p53* knockdown rescues defective elimination and loss of mTOR activity during competition.

A. Knockdown efficiency of p53 siRNA in *Bmrp1a*$^{-/-}$ cells was assessed by analysing mRNA levels of p53 and target p21 by qPCR. B. Fold change in *Bmpr1a*$^{-/-}$ cell number from d0-d5 in cells cultured alone or with wild-type cells when transfected with a control siRNA or an siRNA targeting p53. C. Quantification of flow cytometry staining of pS6$^{S240/244}$ levels in control or p53-knockdown cells at day 3 in competition. D. Knockdown efficiency of p53 siRNA in 4n cells was assessed by analysis of mRNA levels of p53 by qPCR. E. Fold change in 4n cell number from end of assay relative to initial seeding number in cells cultured alone or with wild-type cells when transfected with a control siRNA or an siRNA targeting p53. F. Quantification of median fluorescence of pS6$^{S240/244}$ in 4n cells relative to wild-type cells when transfected with a control siRNA or an siRNA targeting p53 in separate and co-culture at day 3 in competition assessed by flow cytometry. G. Two independent *Bmpr1a*$^{-/-}$;*p53*$^{-/-}$ clones targeted by CRIPSPR were picked for further analysis. These had lost p53 expression as revealed by western blot analysis. n=3 for all studies. Error bars denote SEM. *p<0.05, **p<0.01 and *** p<0.005; unpaired, two-tailed *t*-test (A, E) or ANOVA and Tukeys post-hoc test (B,C,F,G).

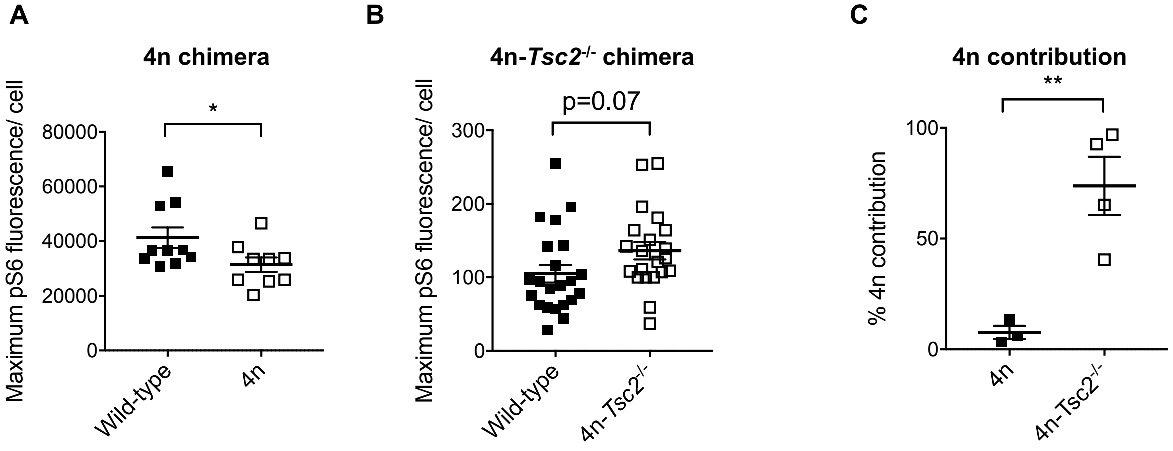

Supplementary Figure 7. Mutation of *Tsc2* rescues pS6 levels in 4n chimeras.

A. Analysis of pS6 levels detected by immunofluorescence in single cells from 4n/wild-type chimeras and (B) 4n-*Tsc2*$^{-/-}$/wild-type chimeras. C. Percentage contribution of 4n and 4n-*Tsc2*$^{-/-}$ cells to wild-type embryos was quantified. *p<0.05, **p<0.01; unpaired, two-tailed *t*-test.

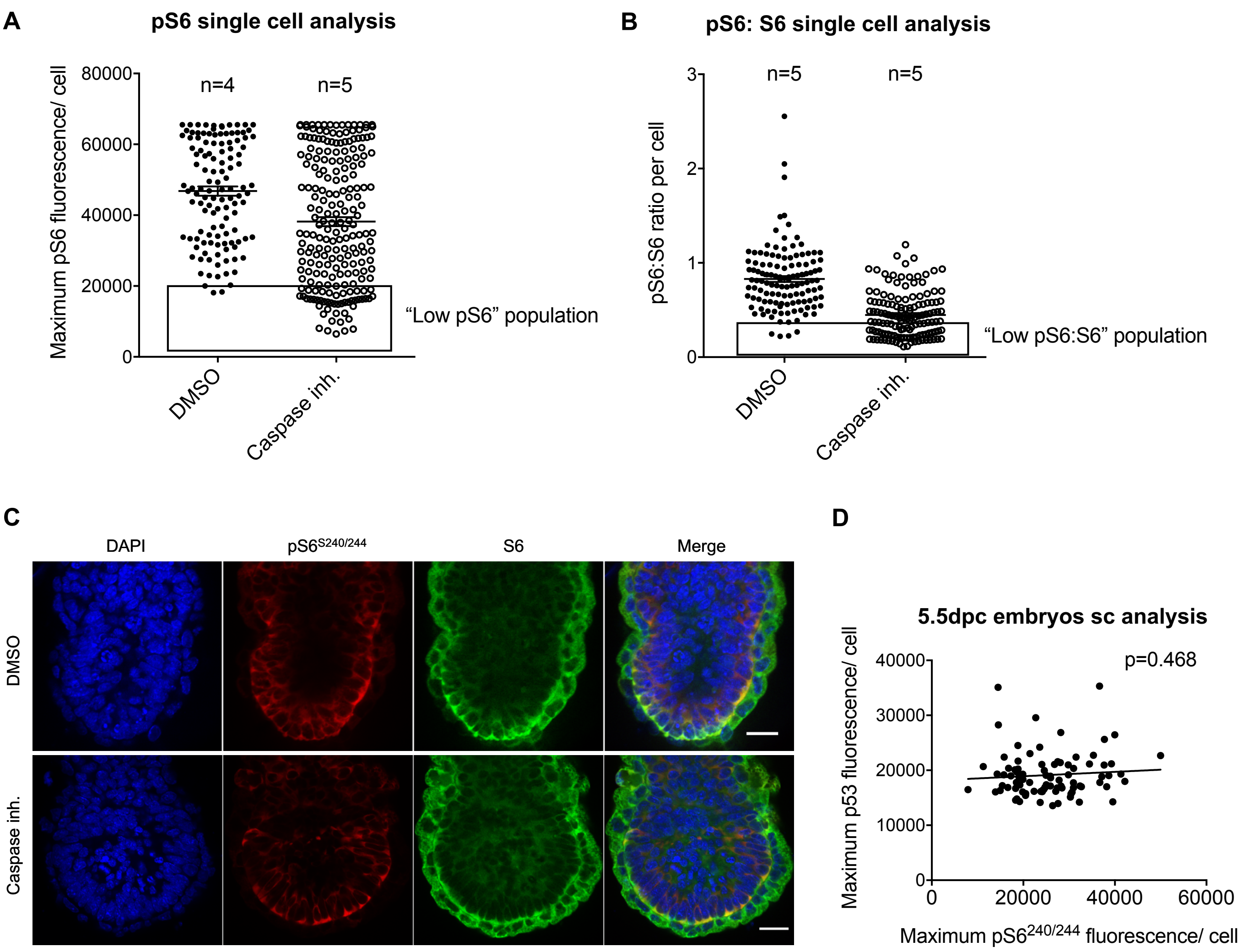

Supplementary Figure 8. Further analysis of the levels of pS6 and p53 in the early mouse embryo.

A. DMSO- or caspase inhibitor- treated embryos were stained for pS6 or (B) both pS6 and total S6 and levels of pS6 or pS6:S6 ratio in each epiblast cell of the embryo was quantified. The box indicates cut-off used to assign cells with low pS6 or low pS6:S6 levels. Data shown are from 1 litter (A: DMSO n=4, caspase inhibitor n=5; B: DMSO n=5, caspase inhibitor n=5). C. Immunofluorescence staining for total and phospho S6 levels in wild-type embryos cultured overnight with DMSO or a caspase inhibitor. D. Correlation at the single cell level of p53 and pS6 levels detected by immunofluorescence in 5.5dpc wild-type embryos. Scale bar= 25μm.

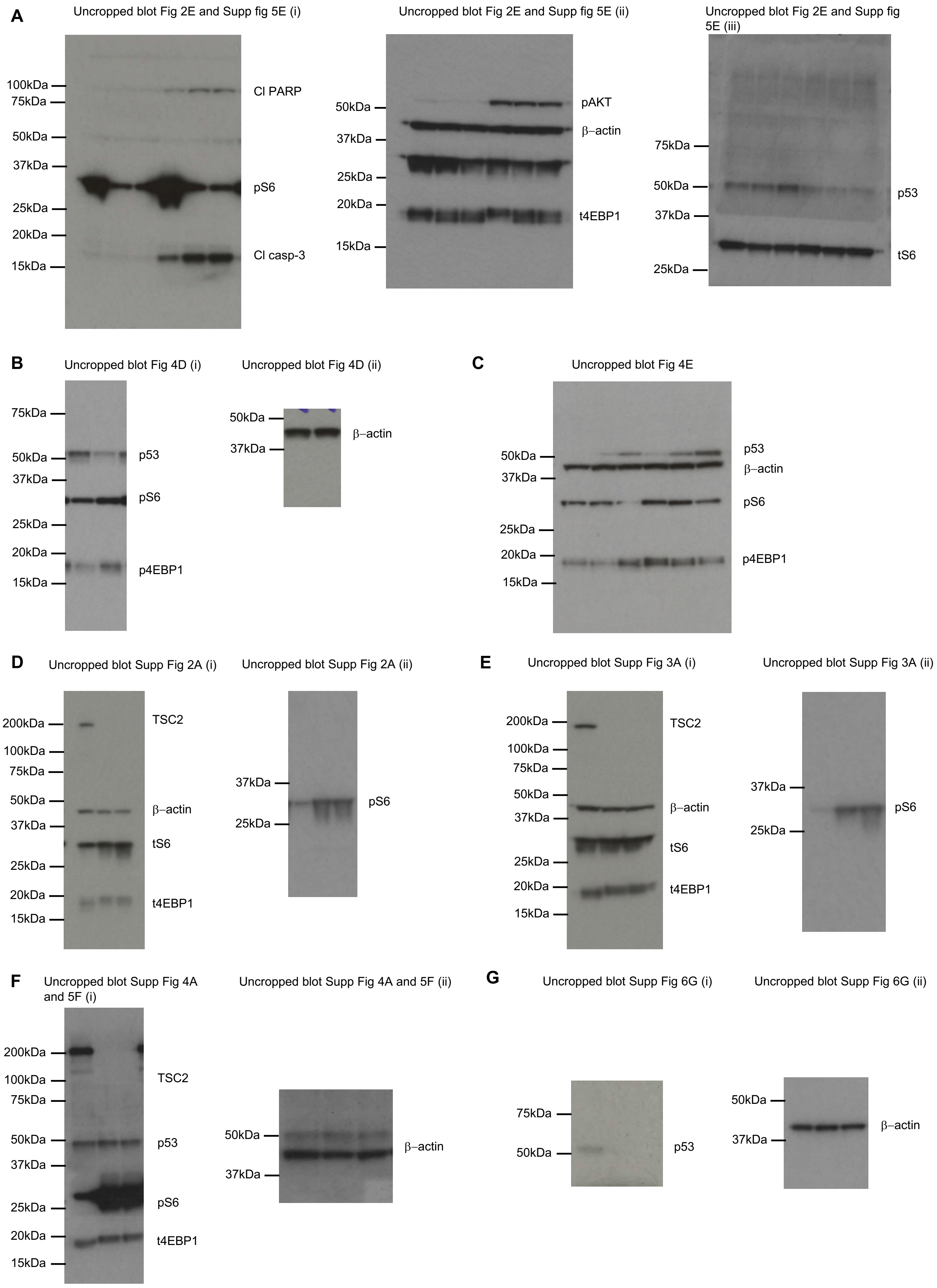

Supplementary Figure 9. Uncropped Western blots
